# The Dynamic Relationship between the Intention and Final Decision for the COVID-19 Booster: A Study among Students and Staff at the University of Liège, Belgium

**DOI:** 10.3390/vaccines10091485

**Published:** 2022-09-06

**Authors:** Marine Paridans, Justine Monseur, Anne-Françoise Donneau, Nicolas Gillain, Eddy Husson, Dieudonné Leclercq, Christelle Meuris, Gilles Darcis, Michel Moutschen, Claude Saegerman, Laurent Gillet, Fabrice Bureau, Michèle Guillaume, Benoit Pétré

**Affiliations:** 1 Public Health Department, Liège University, 4000 Liège, Belgium; 2 Biostatistics Unit—Public Health Department, Liège University, 4000 Liège, Belgium; 3 Infectious Diseases Department, Centre Hospitalier Universitaire de Liège, 4000 Liège, Belgium; 4 Fundamental and Applied Research for Animal and Health (FARAH) Center, Liège University, 4000 Liège, Belgium; 5 Laboratory of Immunology-Vaccinology, FARAH, Liège University, 4000 Liège, Belgium; 6 Laboratory of Cellular and Molecular Immunology, GIGA Institute, Liège University, 4000 Liège, Belgium

**Keywords:** COVID-19, intention and final COVID-19 booster status, academic population, prevention, public health

## Abstract

While many studies have documented the intentions for the COVID-19 vaccine booster, few have explored the change from intention to final decision. This study explores the COVID-19 booster intentions and the change from intention to decision in a primo-vaccinated university population, with a distinction between staff members and students. It looks at the sociodemographic and medical characteristics, health literacy, personal COVID-19 infection and vaccination history, and attitudes/intentions regarding the booster, among the 1030 participants (64.4% staff members, 61.3% female, median age 36.0 years). Of the 8.7% who were initially hesitant, 72.7% ultimately got a booster and 27.3% did not. Another 84.2% intended to get a booster and 7.1% did not. Among the latter two groups, 88.9% maintained their intention and 11.1% changed their minds. The determinants associated with the intentions were health literacy and previous intentions regarding the COVID-19 primo-vaccination. The determinants associated with the change to non-vaccination were a previous COVID-19 infection, a past COVID-19 primo-vaccination intention, and a neutralizing antibody level. The results point to an opening for the support in decision-making, with a significant percentage of the study population potentially changing their mind between intention and final decision; this process should start early and be tailored to the individual’s COVID-19 history. A personalized approach seems necessary in order to ensure that individuals make an informed choice.

## 1. Introduction

Since 2020, the COVID-19 pandemic has swept the globe, bringing tragic consequences for the population and prompting government and public health officials to consider actions in order to limit the spread and transmission of the virus [1]. Of all of the measures aimed at monitoring and controlling the pandemic’s spread, a vaccine development appeared to be key. On 21 December 2020, the European Commission gave its official marketing approval for the first vaccine [2] and on 27 December 2020, vaccination campaigns began, based initially on prioritizing target populations before offering the vaccine to the general population [3,4]. The primary vaccination demonstrated a strong protection against the COVID-19 infection, hospitalization, and death [5]. Information about the declining effectiveness in preventing an infection and severe illness [6] and the limited protection against variants [7] pointed to the need for an additional vaccine dose or booster dose (i.e., a third dose for two-dose regimens and a second dose for single-dose regimens). Hence, the first booster campaigns (mRNA vaccines only) began in late July 2021 [8]. Those with a lowered immunity (mainly the elderly and health workers in some countries) were invited for a second booster dose starting in early 2022 [9]. The recent data on the vaccination coverage around the world showed that 65.1% of people had received at least one dose, 59.0% had been fully vaccinated (primary vaccination), and 23.8% had received a COVID-19 booster by early May 2022 [10]. While the data at time of this writing shows that booster shots do raise the immune response, recent data on the booster’s effectiveness against variants and its longer-term effect on immunity are more controversial [11].

Although the primary vaccination rates in Europe were relatively high, the data on the vaccination intentions showed them fluctuating. For example, a joint study by IPSOS and the World Economic Forum showed changes in the vaccination intentions in August 2020 and between December 2020 and February 2021. Among the 15 countries surveyed, intentions rose significantly in Italy and reached 62% in February 2021 (25% higher than in August 2020 and 36% higher than in December 2020), as compared to Russia, where intentions at the end of February 2021 was 16% (3% lower than in August 2020 and 2% higher than in December 2020) [12]. These trajectories clearly show that a vaccination intention against COVID-19 changes over time and should therefore be considered a dynamic process. Although the COVID-19 crisis is not yet over and its course remains uncertain, the heightened and predictable threat of new pandemics requires reflection about a long-term prevention strategy—and hence a better understanding of how a vaccination intention works.

The determinants of a vaccine hesitancy are discussed in the literature. They can be grouped in a variety of ways, e.g., by contextual influences, by individual and group influences, by vaccine/vaccination-specific issues [13], or by confidence, complacency, constraints, calculation, or collective responsibility [14]. Regarding the COVID-19 vaccination in particular, a systematic search of the peer-reviewed literature shows that safety, side effects, effectiveness, trust, information sufficiency, efficacy, conspiracy beliefs, social influence, political roles, vaccine mandates, and fear and anxiety were determinants of the COVID-19 primo-vaccination [15]. Among these multiple determinants, a personal vaccine history (i.e., past events that discouraged the individual from getting a vaccine or past experience with a vaccination [13]; duration of the induced immunity; the type of vaccine); a loss of confidence in the vaccination policy; and general pandemic fatigue [16] appear to be the major factors regarding the COVID-19 booster vaccination when it is part of a multiple-shot vaccination regimen using a new vaccine over a short period of time.

A number of studies have looked at the COVID-19 vaccine booster intentions in different countries and in different populations. Some have shown that a younger age; a lower educational level; better health; a mistrust of authorities, the government, and the pharmaceutical industry; an initial uncertainty; and an unwillingness to get the first COVID-19 vaccine were associated with a higher vaccine hesitancy or unwillingness to get a booster [17,18,19,20,21]. These studies showed the potential determinants of the COVID-19 booster intentions but, to the best of our knowledge, provided no information on which factors might influence a change between intention and the final decision concerning the booster. Having a better understanding of the dynamics between intention and action with regards to the COVID-19 booster is hugely important from a public health standpoint.

In Belgium, little is known about the COVID-19 booster intentions or about the change between the initial intention and final decision concerning a COVID-19 booster, in particular. Prior to the COVID-19 vaccination campaign, a survey conducted among 2060 adults in Belgium, between 6 and 16 October 2020, showed that 73% declared that they intended to get vaccinated—34% definitely and 39% probably. This intention was associated with age, opinion on the government’s dealing with the COVID-19 pandemic, medical risk, spoken language, gender, and having known someone who was hospitalized because of COVID-19 [22]. The COVID-19 vaccination program began on a voluntary basis in early 2021 and proceeded in several phases: (1) health professionals and residents of nursing homes; (2) at-risk people and the elderly; and (3) the general population. Two types of vaccines have been approved: mRNA vaccines (PfizerBioNTech and Moderna), and viral vector vaccines (AstraZeneca and Johnson & Johnson) [23]. According to Sciensano, 75.4% had been fully vaccinated (received doses 1 and 2) against COVID-19 and 76.7% only partially vaccinated (received dose 1) by the end of November 2021. Between April and July 2021, the risk of infection fell by 80–90% amongst the fully vaccinated compared with the unvaccinated. In addition, from the start of the campaign until the end of October 2021, an estimated 30,000 hospitalizations were avoided thanks to the vaccination campaign [24]. The strategy for COVID-19 booster vaccinations, launched on a large scale on 27 November 2021 and once priority groups (the elderly, immunocompromised, and healthcare workers) had been boosted, is based on the amount of time since the previous vaccine dose and on age. An mRNA vaccine (Moderna or PfizerBioNTech) was recommended, regardless of which type of vaccine was used for the primary vaccination [25]. Immune compromised people were invited to receive a second booster dose in the spring of 2022 [26]. According to Sciensano, 80.2% of Belgians aged 18 years and over received at least one dose, 79.4% were fully vaccinated (primary vaccination), 61.9% received one booster dose, and 2.08% received a second booster dose by early May 2022 [27].

In this context, the aim of this article is two-fold:

- to describe the initial vaccination intention regarding the COVID-19 booster in a university population and associated factors; and
- to describe the change between the initial vaccine intention and the final decision regarding the COVID-19 booster dose and the factors associated with that change in the same population.

## 2. Materials and Methods

### 2.1. Context

This research is part of a longitudinal study conducted among students and staff members at the University of Liège (ULiège), Belgium between April 2021 and June 2022, which aims to study COVID-19 infections, the immune responses to COVID-19 infections and vaccines, and vaccine hesitancy (SARSSURV-ULiège study). The inclusion criteria were as follows: the participants had to be between 18 and 67 years of age (67 corresponding to the age of retirement specified by law in Belgium), and to have given their consent to participate via an online form. Any staff member whose contract ended before 31 June 2022 and any students enrolled in the 2021–2022 degree year were excluded [28]. 

### 2.2. Study Population 

The population of ULiège includes 5633 staff members and 28,064 students. All university members who met the study criteria received a personalized invitation to participate in the SARSSURV study, namely 3576 staff members and 25,378 students. At the time of the booster study, 1320 participants (835 staff members and 485 students) were still enrolled in the SARSSURV study.

**Figure 1:**
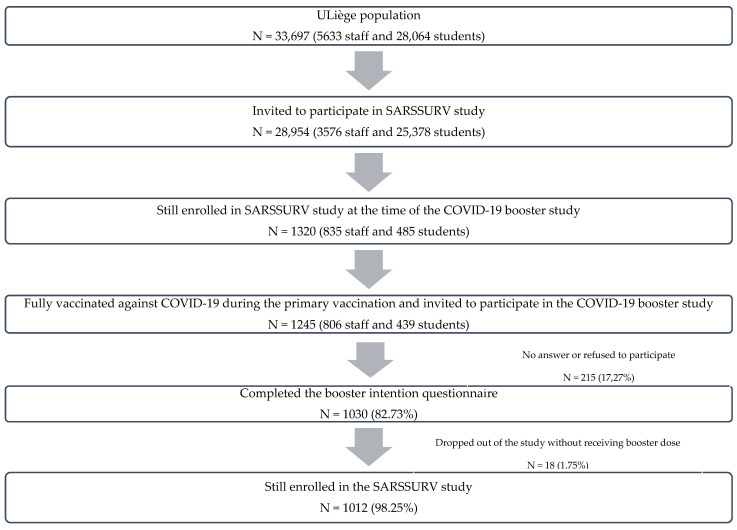
Flowchart of booster study participants.

Based on the SARSSURV-ULiège database, the survey’s target population was students and staff members who were fully vaccinated against COVID-19 during the primary vaccination (one dose of Johnson & Johnson or two doses of AstraZeneca, Moderna, PfizerBioNTech, or Sputnik V) and who volunteered to participate in this study. A total of 1245 members of the University of Liège community (806 staff members and 439 students) received a personalized invitation, sent via the university’s internal mail system, to participate in the booster survey. Of these, 1030 completed the survey on a booster intention, with a response rate of 82.7%. Of those 1030 participants, 18 (1.8%) dropped out of the study without receiving a COVID-19 booster and 1012 (98.3%) were still enrolled in the study (Figure 1). Finally, 1030 participants were included in the analyses related to the intention to vaccinate and 1012 in the analyses related to changes between intention and final decision regarding the COVID-19 booster vaccination.

### 2.3. Studied Parameters and Data Collection

The data were collected from several sources at various times during the SARSSURV study. First, the sociodemographic characteristics, medical characteristics, health literacy, and COVID-19 infection and vaccination history were collected using the self-administered questionnaires distributed via an online platform during the study registration period, starting on 1 April 2021. Next, throughout the SARSSURV study, the participants were invited to provide (via the online platform or by calling/emailing a member of the research team) information regarding any change in their vaccination status or a COVID-19 infection, as soon as possible, in order to keep the database up-to-date. Following any new infection or vaccination, a nurse from the research team scheduled a blood draw at two weeks and then at every three months, and collected the data concerning symptoms via a short questionnaire. Third, specifically regarding the COVID-19 booster vaccination (the perception of and intention to get a COVID-19 booster), the study used a self-administered questionnaire that was distributed via an online platform. The data were collected between 13 October 2021 and 26 December 2021 [28].

### 2.4. Sociodemographic Characteristics

The sociodemographic characteristics included the institutional status (response scale: student or staff member), gender (response scale: male or female), age (response scale: open ended response in years) and highest level of education (response scale: no degree, primary education, lower secondary education, upper secondary education, post-secondary non-tertiary education, short-type higher education, long-type higher education (University), and PhD).

### 2.5. Medical Characteristics

The body mass index (BMI, response scale: open ended response in kg/m^2^), chronic diseases such as diabetes, hypertension, heart failure/coronary artery disease, history of stroke, liver failure/cirrhosis, kidney failure, chronic lung disease, asthma, autoimmune disease, immunodeficiency, hematologic cancer, other cancer, organ or cell transplant, and other health problem(s) (response scale: yes/no) were collected.

### 2.6. Health Literacy

The single item literacy screener (SILS), slightly adapted for this study, was used in order to assess the health literacy of ULiège staff members and students [29]. “When you read instructions, pamphlets, or other written material from your doctor or pharmacy, how often do you need help to understand the messages?” (response scale: Likert scale ranging from 0 (never) to 100 (always)).

### 2.7. Personal COVID-19 Infection and Vaccination History

The COVID-19 infection history included COVID-19 infections (yes/no), confirmed by a saliva-based self-test performed as part of the SARSSURV study [28] or by a test carried out outside of the study and reported by a research team participant (saliva test, nasopharyngeal test, self-test). 

The COVID-19 vaccination history examined the participants’ past intention to be vaccinated against COVID-19 using the following two questions “On a scale of 0 to 100, what was your intention to get vaccinated at the end of 2020?” (response scale: Likert scale ranging from 0 (no intention) to 100 (total intention)) and “On a scale of 0 to 100, what is your current (April 2021) intention to get vaccinated when a vaccine is offered?” (response scale: Likert scale ranging from 0 (no intention) to 100 (total intention)). In addition, the participants were asked which type of vaccine they received for dose 1 and dose 2—response scale: AstraZeneca, Moderna, PfizerBioNTech, Johnson & Johnson or “I don’t know”—and about any symptoms they experienced after the last dose, e.g., fatigue, headache, appetite loss, muscle pain, delirium, nausea, vomiting, fever, arthralgia (joint pain), injection site pain, ipsilateral axillary lymphadenopathy (swollen lymph node(s) on the same side as the injection site), redness at the injection site, allergic reaction, and others (response scale: Likert scale ranging from 0 (no symptoms) to 10 (severe symptoms)).

The level of the neutralizing antibodies against COVID-19 was collected using the most recent blood sample, which was scheduled 15 days after a positive COVID-19 infection or a COVID-19 vaccination and then every three months as follow-up. A member of the research team communicated the results to each participant via phone or letter.

### 2.8. Perceptions of the COVID-19 Booster Vaccination

The participants were asked about their primary reason for accepting or refusing the COVID-19 vaccine booster with the following two questions: “What is the main reason you would get a COVID-19 vaccine booster?” (response scale: open ended response) and “What is the main reason why you would refuse to get a COVID-19 vaccine booster?” (response scale: open ended response).

### 2.9. Intention Regarding the COVID-19 Booster Vaccination

The participants’ intentions regarding the COVID-19 booster vaccination were collected with the question, “What is your current intention regarding accepting a booster dose when offered? (response scale: resistance, hesitance, or acceptance)”.

### 2.10. COVID-19 Booster Vaccination

The participants reported any change in their vaccination status and the date of the vaccination. If participants entered a date for a booster dose, they were considered boosted. Otherwise, they were considered not boosted.

### 2.11. Data Analysis

For the dependent variables, three outcomes were considered. The first outcome was the intentions regarding the COVID-19 booster vaccination (no, hesitancy, yes). For the COVID-19 booster vaccination status, two outcomes were constructed based on the initial vaccination intention and the final decision for the COVID-19 booster (yes/no):

- the change between the intention and decision among the hesitant group, with two response modes: positive change (from hesitant to yes) and negative change (from hesitant to no);
- the change between the intention and decision in those who intended or did not intend to get the booster, with three response modalities: maintenance of the intention (whether negative or positive); a change from a negative intention to getting the booster; and a change from a positive intention to not getting the booster.

The educational data were grouped into three categories (high school and lower, Bachelor’s degree, and University), and all of the positive COVID-19 infections were grouped into a single variable (presence or absence of infection). The number of chronic diseases was calculated. The symptoms experienced after the COVID-19 vaccination were classified as asymptomatic (0), mild symptoms (1), moderate symptoms (2–4), and severe symptoms (≥5) and counted, based on the tertiles of our sample. The reasons for accepting or refusing the COVID-19 booster were categorized based on the qualitative responses: self-protection and protection against variants/sufficient protection; protecting others/does not contribute to a herd immunity; compliance with the vaccination strategy/disagreement with the vaccination strategy; possible vaccination side effects; geographical and financial (in)accessibilities; (mis)trust in the sources of information, (lack of) efficacy; contraindication; and no reason. 

The histograms, quartile plots, and the Shapiro–Wilk tests were used in order to evaluate whether the distribution of the quantitative variables was Gaussian. The descriptive statistics were performed, with the frequency and percentage (%) used to report the qualitative variables and median and the interquartile ranges (P25–P75) were used to report the quantitative variables, due to any non-normality. The comparisons between staff and students were made with the unpaired t test or Mann–Whitney for the quantitative variables and with the χ^2^ test and Fisher’s exact test for the categorical variables. The characteristics of the staff and students were different and the analyses were carried out for each of the groups.

The univariate logistical regressions were performed in order to explore the relationship between the intention regarding the COVID-19 booster vaccination and the various factors related to the sociodemographic characteristics, medical characteristics, health literacy, history of a COVID-19 infection, and primo-vaccination. The multivariate logistical regression models were created by entering all of the variables significantly associated with the intention regarding the COVID-19 booster vaccination into a forward stepwise logistical regression. The interaction analyses between the significant variables were also performed. The results of the interaction analyses were presented in Supplementary Materials for further reference. The same statistical methodology was used in order to analyze the final vaccination status. The calculation of the areas under the ROC curve was used as an indicator of the quality of a model [30]. The significance level was set at *p* < 0.05. The analyses were performed using SAS (version 9.4 for Windows) and R statistical software. The data are stored for as long as necessary in order to achieve the study’s objectives. Due to the number of participants who dropped out of the SARSSURV study after completing the questionnaire, the analyses related to the vaccination intention and those related to the final vaccination status were not performed on the same amount of data. The statistical analyses were performed on the observed data only; the missing data were not replaced. 

### 2.12. Ethical and Legal Aspects

The study was approved by the University Hospital of Liège Ethics Committee (reference number 2021/96, dated 26 March 2021). The informed consent was obtained prior to the enrollment in the SARSSURV study. After the enrollment in the SARSSURV study, a unique identification code (ID) was attributed to each participant [28]. The data were handled in a confidential manner by the SARSSURV team and anonymized prior to any analysis. The compliance with data protection regulations were approved by the official University of Liège data protection officer.

## 3. Results

### 3.1. Characteristics of the Study Sample 

As shown in Table 1, of the 1030 participants included in this study, 64.4% were staff members and 61.3% were female. The median age of participants was 36.0 years (44.0 years for staff and 23.0 years for students); 59.7% (73.0% of staff and 35.7% of students) had a university-level education, 20.0% (23.7% of staff and 13.4% of students) had a Bachelor’s degree, and 20.3% (3.3% of staff and 51.0% of students) had a high school or lower level of education. Regarding the health status, the median participant’s BMI was 23.4 kg/m^2^ (24.1 kg/m^2^ for staff and 22.3 kg/m^2^ for students) and the median number of chronic diseases was zero for staff and students. The participants reported a median health literacy score of 7.0 (7.0 for staff and 9.0 for students) on a Likert scale ranging from 0 (never) to 100 (always). Regarding COVID-19, a minority of participants had a previous COVID-19 infection: 23.5% (24.4% of staff and 21.8% of students) prior to completing the dose booster questionnaire and 33.9% (32.5% of staff and 36.4% of students) prior to the booster vaccination. The median past intention regarding the COVID-19 vaccination was 90.0 (90.0 for staff and 85.0 for students) at the end of 2020 and 100 (staff and students) at the time of enrollment in the study (since April 2021). The majority of the participants received the PfizerBioNTech vaccine for their first and second doses (76.0%–70.2% of staff and 86.6% of students and 78.6%–73.7% of staff and 87.3% of students, respectively), followed by the AstraZeneca vaccine (13.7%–19.2% of staff and 3.8% of students and 13.8%–19.4% of staff and 3.9% of students, respectively), the Moderna vaccine (7.2%–6.3% of staff and 8.8% of students and 7.5%–6.7% of staff and 8.8% of students, respectively) and the Johnson & Johnson vaccine (3.0%–4.2% of staff and 0.8% of students and 0.1%–0.2% of staff and zero students, respectively). Due to the distribution of the vaccine type variable, this variable was not included in the univariate and multivariate analyses. Following the COVID-19 vaccination, 45.3% of participants (43.5% of staff and 49.0% of students) experienced moderate symptoms, 22.5% (24.1% of staff and 18.9% of students) experienced mild symptoms, 20.5% (18% of staff and 26.0% of students) experienced severe symptoms, and 11.7% (14.4% of staff and 6.1% of students) were asymptomatic. Blood was taken from 773 participants (607 staff members and 166 students) before completing the booster dose questionnaire and from 962 participants (641 staff members and 321 students) before receiving the booster dose. The median neutralizing antibody titers were 40 (40 for staff and 80 for students) and 80 (40 for staff and 80 for students), respectively.

### 3.2. Evolution between Intention Regarding the COVID-19 Booster Vaccination and Final Decision Concerning the Booster Vaccination

Overall, 84.2% of participants (87.8% of staff and 77.7% of students) intended to get the COVID-19 booster vaccination, 8.7% (7.2% of staff and 11.4% of students) were hesitant, and 7.1% (5.0% of staff and 10.9% of students) did not intend to get the booster. 

The perceptions of the COVID-19 booster vaccination are shown in Table 2. Regarding the main reason for accepting the COVID-19 booster vaccination, 55.4% of participants (58.8% of staff and 49.3% of students) cited self-protection, 21.5% (22.6% of staff and 19.3% of students) cited protecting others, and 16.8% (13.9% of staff and 22.1% of students) cited compliance with the vaccination strategy. Regarding the main reason for refusing the COVID-19 booster vaccination, 34.4% of participants (35.1% of staff and 33.0% of students) did not cite any reason, 23.8% (25.5% of staff and 20.7% of students) cited harmfulness, 11.0% (9.0% of staff and 14.4% of students) cited disagreement with the vaccine strategy, and 10.8% (11.8% of staff and 9.0% of students) cited a lack of efficacy.

**Figure 2:**
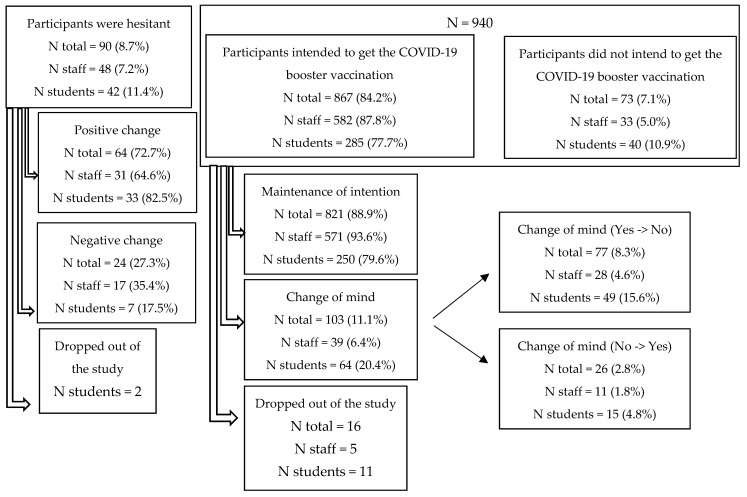
Actual booster dose status of the 1012 participants according to their original intention.

Finally, of the participants who were initially hesitant (*n* total = 88; *n* staff = 48; *n* students = 40), 72.7% (64.6% of staff and 82.5% of students) got the booster and 27.3% (35.4% of staff and 17.5% of students) did not. Among the participants who had either intended to get the booster or did not intend to get the booster (*n* total = 924; *n* staff = 610; *n* students = 314), 88.9% (93.6% of staff and 79.6% of students) followed through on their intention and 11.1% (6.4% of staff and 20.4% of students) changed their minds and 8.3% (4.6% of staff and 15.6% of students) who had intended to get the booster dose chose not to and 2.8% (1.8% of staff and 4.8% of students) of those who did not intend to get the booster later decided to get it (Figure 2).

### 3.3. Factors Influencing the Intention to Get the COVID-19 Booster

The univariate logistical regression showed that the health literacy and past COVID-19 vaccination intention were significantly associated with the intention regarding the COVID-19 booster vaccination among staff members. The participants with a lower past COVID-19 vaccination intention were more likely to refuse the booster dose or be hesitant than those with a higher past COVID-19 vaccination intention (*p* < 0.05), who were more likely to get the booster. Compared with staff members with a higher health literacy, and who were more likely to get a booster dose, those with a lower health literacy were more likely to be hesitant (*p* < 0.05).

The multivariate forward stepwise logistical regression found that a past COVID-19 vaccination intention was a determinant of the booster vaccination intention among staff members. The participants with a higher COVID-19 vaccination intention at the end of 2020 (*p* < 0.05) were more likely to refuse the booster dose. In addition, the participants with a higher COVID-19 vaccination intention at the time of the SARSSURV study enrollment were less likely to refuse the booster dose or be hesitant about it (*p* < 0.05) than the participants with a lower COVID-19 vaccination intention, who were more likely to refuse the booster dose or be hesitant about it (Table 3). The calculation of the areas under the ROC curve was 0.73.

The univariate logistical regression showed that a health literacy and past COVID-19 vaccination intention were significantly associated with the intention regarding the COVID-19 booster vaccination among students. Compared with students with a higher health literacy, who were more likely to get a booster dose, those with a lower health literacy were more likely to be hesitant (*p* < 0.05). The participants with a lower past COVID-19 vaccination intention were more likely to refuse the booster dose or be hesitant than those with higher past COVID-19 vaccination intention (*p* < 0.05), who were more likely to get the booster. 

The multivariate forward stepwise logistical regression found that a health literacy and past COVID-19 vaccination intention was a determinant of the booster vaccination intention among students. Compared with students with a higher health literacy, who were more likely to get a booster dose, those with a lower health literacy were more likely to be hesitant (*p* < 0.05). The participants with a higher COVID-19 vaccination intention at the end of 2020 were less likely to refuse the booster dose or be hesitant about it (*p* < 0.05) than the participants with a lower COVID-19 vaccination intention, who were more likely to refuse the booster dose or be hesitant about it. In addition, the participants with a higher COVID-19 vaccination intention at the time of the SARSSURV study enrollment were less likely to refuse the booster dose (*p* < 0.05) than the participants with a lower COVID-19 vaccination intention, who were more likely to refuse the booster dose (Table 4). The calculation of the areas under the ROC curve was 0.73.

### 3.4. Factors Influencing the Change between the Intention and Final Decision regarding the COVID-19 Booster

The univariate logistical regressions and multivariate forward stepwise logistical regression of factors influencing the change between the intention and final decision regarding the COVID-19 vaccine booster among the hesitant staff members are shown in Table 5. The univariate analyses showed that a COVID-19 infection and the neutralizing antibody levels were significantly associated with the change between the intention and final decision regarding the COVID-19 booster. The participants who got COVID-19 were less likely to be vaccinated than the participants who had not caught COVID-19 (*p* < 0.05). The higher the neutralizing antibody levels, the less likely the participants were to get vaccinated (*p* < 0.05). Following the multivariate analysis, only the COVID-19 infection variable was significantly associated with the change between intention and the final decision regarding the COVID-19 booster. The participants who had been infected were less likely to get vaccinated than were the participants who had not been infected (*p* < 0.05). The calculation of the areas under the ROC curve was 0.80.

The univariate logistical regressions and multivariate forward stepwise logistical regression of factors influencing the change between the intention and final decision regarding the COVID-19 vaccine booster among the hesitant students are shown in Table 6. The univariate analyses found no significant variable.

The univariate logistical regressions and multivariate forward stepwise logistical regression of the factors influencing the change between the intention and final decision regarding the COVID-19 booster vaccination among staff members who changed their mind are shown in Table 7. The univariate analyses showed that a COVID-19 infection, a past COVID-19 vaccination intention and neutralizing antibody levels were significantly associated with a change between the intention and final decision regarding the COVID-19 booster. The participants who got COVID-19 were more likely to change their mind (yes->no) (*p* < 0.05) than the participants who had not caught COVID, who were more likely to carry out their original intention. The higher the past vaccination intention, the less likely the participants were to change their minds (no->yes: *p* < 0.05; yes->no: *p* < 0.05). The higher the neutralizing antibody levels, the more likely the participants were to change their mind (yes->no) (*p* < 0.05). 

The multivariate analysis showed that a COVID-19 infection, a past COVID-19 vaccination intention at enrollment in the SARSSURV, and the neutralizing antibody levels were significantly associated with a change between the intention and final decision regarding the COVID-19 booster vaccination. The participants who got COVID-19 were more likely to change their mind (yes->no) (*p* < 0.05) than the participants who did not get COVID-19, who were more likely to follow through on their intention. The higher the past vaccination intention at the study enrollment, the less likely the participants were to change their mind (no->yes) (*p* < 0.05). The higher the neutralizing antibody levels, the more likely the participants were to change their mind (yes->no) (*p* < 0.05). The calculation of the areas under the ROC curve was 0.79.

The univariate logistical regressions and multivariate forward stepwise logistical regression of the factors influencing the change between the intention and final decision regarding the COVID-19 booster vaccination among students who changed their mind are shown in Table 8. The univariate analyses showed that a COVID-19 infection, a past COVID-19 vaccination intention at the end of 2020 and the neutralizing antibody levels were significantly associated with a change between the intention and final decision regarding the COVID-19 booster. The participants who got COVID-19 were more likely to change their mind (yes -> no) (*p* < 0.05) than were the participants who had not caught COVID, who were more likely to carry out their original intention. The higher the past vaccination intention at the end of 2020, the less likely the participants were to change their mind (no->yes) (*p* < 0.05). The higher the neutralizing antibody levels, the more likely the participants were to change their mind (yes->no) (*p* < 0.05).

The multivariate analysis showed that a past COVID-19 vaccination intention at the end of 2020, and the neutralizing antibody levels were significantly associated with a change between the intention and final decision regarding the COVID-19 booster vaccination. The higher the past vaccination intention at the end of 2020, the less likely the participants were to change their mind (no->yes) (*p* < 0.05). The higher the neutralizing antibody levels, the more likely the participants were to change their mind (yes->no) (*p* < 0.05). The calculation of the areas under the ROC curve was 0.72.

## 4. Discussion

To the best of our knowledge, this is the first study in Belgium to compare the factors associated with the COVID-19 vaccine booster intention and the changes between intention and final decision regarding the COVID-19 booster, in a fully primo-vaccinated academic population, namely staff and students at the University of Liège. Indeed, rare are the articles that offer an original and dynamic approach to the intention and vaccination with a view to understanding such changes.

The results show that of the 1030 University of Liège staff members and students who participated in the study, 84.2% (87.8% of staff and 77.7% of students) intended to get the COVID-19 booster vaccination, 8.7% (7.2% of staff and 11.4% of students) were hesitant, and 7.1% (5.0% of staff and 10.9% of students) did not intend to get it. These results are comparable with a study involving German university students (*n* = 608) and employees (*n* = 322), which found that 87.8% (90.7% of employees and 86.3% of students) intended to get the booster, 4.4% (5.6% of employees and 3.8% of students) were hesitant, and 7.7% (3.7% of employees and 9.9% of students) did not intend to get it [31]. These results are also found in the general population, but with very marked differences according to different age groups. For example, a survey conducted among 1256 adults aged 18 and over in France, between November 30 and December 7, 2021, showed that 89% declared that they intended to get the booster dose—65% certainly and 24% probably. However, this intention was 66% among 25 to 34-year-olds and 90% among those age 65 and over [32]. The vaccine acceptance was relatively high and motivated by a number of factors—also found by other studies in university [31,33] and general populations [19,34,35]—such as self-protection and protection of others. However, harmfulness, disagreement with the vaccine strategy, and lack of information and efficacy are the main reasons for a COVID-19 vaccine booster hesitancy or refusal. These results were also found by other studies [17,20,33,34,35,36,37,38]. Hence, the COVID-19 vaccination/booster campaigns should provide clear information on the efficacy and safety of the COVID-19 vaccines.

Regarding the factors influencing the COVID-19 booster intention, a lower health literacy was a determinant of the vaccine hesitancy among students and a higher COVID-19 vaccination intention at the end of 2020 was a determinant of the non-intention among staff members but a determinant of the intention among students. In addition, a COVID-19 vaccination intention during the SARSSURV study enrollment was a determinant of the intention. These results are found in other studies. For example, one study, in the UK, showed that an initial hesitancy and unwillingness to get the first COVID-19 vaccine in 2020–2021 were associated with the uncertainty about and unwillingness to get a vaccine booster [21]. In addition, a study in the United States that used a validated health literacy questionnaire in order to assess the vaccine literacy via 14 questions and divided into three scales (functional, interactive, and critical) showed that the hesitant group had lower scores than the non-hesitant group [20]. Hence, a positive intention was not always sustainable over time, which implies that support strategies are needed throughout the vaccination campaign. In addition, there is a need to build health skills among students in particular in order to help them make an informed choice in terms of the COVID-19 vaccination.

Our study goes beyond the results of previous studies to show the change in intention regarding the COVID-19 vaccine booster. Of the participants who definitely intended to get the booster dose or definitely did not intend to get the booster dose, 88.9% (93.6% of staff and 79.6% of students) carried out their intention and 11.1% (6.4% of staff and 20.4% of students) changed their minds. Of the participants who were hesitant about the booster, 72.7% (64.6% of staff and 82.5% of students) ultimately got the booster and 27.3% (35.4% of staff and 17.5% of students) did not. While the majority of the participants carried out their initial intention, a positive intention did not completely determine a follow-through, implying that support strategies are needed both before vaccination campaigns and throughout the vaccination process in order to help individuals make an informed choice in terms of the COVID-19 vaccination.

Our results show that some factors negatively influenced the change between intention and final decision regarding the COVID-19 booster vaccination. A higher past vaccination intention (at enrollment of the study for staff and at the end of 2020 for students) was a determinant of continuing to refuse the COVID-19 booster. Having higher neutralizing antibody levels was a determinant of the COVID-19 booster dose refusal among staff and students who initially intended to get boosted, as was the personal experience of a previous COVID-19 infection among staff. While having a low neutralizing antibody level, and a past vaccination intention were found to be determinants of the COVID-19 booster intention by other studies, those studies did not examine any associations with the change from intention to final decision [21,36]. The results regarding a COVID-19 infection showed that previously infected people prior to any vaccination were less likely to get a COVID-19 booster [31,37,38]. 

The change in intention results help identify several key messages:

(1) First, the participants tended to maintain their original intention to get vaccinated against COVID-19. This implies that stronger support strategies are needed both at the start of the vaccination campaigns—given how original intentions tend to predict attitudes toward any subsequent vaccine doses—and throughout the vaccination process;
(2) Second, students seem to be more sensitive to a negative change in their intention to get vaccinated against COVID-19. There are several possible explanations for this: sometimes young adults are less concerned about vaccination for a variety of reasons, such as the misconceptions about natural immunity, the belief in the vaccine risk-benefit ratio, the fear of new vaccines, and mistrust of the pharmaceutical industry [39]. In addition, a later COVID-19 vaccination for Belgian youth and the end of the COVID Safe Ticket in March 2022 may have caused young people to change their minds and not get vaccinated. Indeed, people vaccinated earlier were forced to do the booster dose or risk not having a valid COVID Safe Ticket while the students, who received the primary vaccine later, were less under pressure to get a COVID-19 booster. Furthermore, students, less concerned with the vaccination against COVID-19, may have intended to get vaccinated in order to protect others through reduced transmission. However, the evolution of knowledge on the subject has shown that the vaccine has not made it possible to achieve the desired objective. So, students probably changed their minds about the booster dose. This audience requires greater outreach throughout the vaccination process;
(3) Third, the findings from this study underscore the importance of considering more individual factors such as previous COVID-19 infections and COVID-19 antibody levels in raising the public awareness around the COVID-19 vaccination. Indeed, the participants of our study seemed poorly informed about the importance of the vaccination even if they had been infected or had high neutralizing antibody levels. Previously infected participants and those with higher antibody levels also seemed to change their minds and refuse the booster dose probably because their COVID Safe Ticket was still valid following a recent infection. This poses a challenge, because recent studies have shown that previous infections combined with the vaccination produce a higher level of neutralizing antibodies against COVID-19 [40]. Thus, it would be useful to develop a more personalized approach in interactions between such people and health professionals—such as general practitioners and pharmacists—so that they are able to make an informed decision specific to their particular situation. This perspective also highlights the crucial role of the long-dismissed first line of care in the COVID-19 vaccination strategy and is all the more important as the need for a new booster dose, specific or not to the variants, is currently under discussion [41].

In terms of future research prospects, we need similar studies to be carried out in the general population, in order to develop appropriate public health interventions that better support people in their decision-making process around the COVID-19 vaccination.

This study has some strengths. We were able to go beyond earlier studies and explored the COVID-19 booster intention and the changes from intention to decision. In order to get more detailed data on the participants’ perceptions regarding the booster, however, it would have been interesting to do a second study, just before they got (or decided not to get) the booster, on the motivations for and the deterrents against doing so. Indeed, the omicron variant’s emergence during the booster vaccination period certainly changed people’s perceptions. A COVID-19 infection also stood out as a determinant of the final vaccination status among study participants. University populations, namely students and staff members, offer data from a closed population, which in turn allows action on the factors that influence the intention to vaccinate or the decision to vaccinate in a specific population. In addition, the quality of the logistical models is acceptable [42].

This study also has some limitations. First, because it looked at a volunteer sample from a highly educated university population with a higher proportion of women (staff members and students) in the study than in the university, a high health literacy and a positive attitude toward getting vaccinated, the results may not be representative of the university population and cannot be generalized to the Belgian population at large. Second, the sample was not evenly balanced in terms of institutional status, gender, or educational level. Third, there was a social-desirability bias that may have influenced the results, although the COVID-19 booster vaccination intention questionnaire was administered to participants via an online platform in order to minimize such a bias. Fourth, it would have been useful to question the feelings of individuals in relation to the vaccination experiences of their relatives. Lastly, the data concerning changes in the vaccination status were self-reported by the participants and were not verified.

## 5. Conclusions

In conclusion, a large number of university students and staff members intended to get a COVID-19 booster and did get the booster. This study highlighted how the factors related to the booster dose evolved and showed that having a weaker past intention to get vaccinated, having had a previous COVID-19 infection, and having a higher neutralizing antibody level were determinants of a change toward not getting a COVID-19 booster among staff members. Only having a weaker past intention to get vaccinated and having a higher neutralizing antibody level were determinants of this change among students. These results open up to new perspectives in terms of supporting individuals in their decision-making.

## Figures and Tables

**Table 1:** Characteristics of the 1030 participants included in the study.

Variable	All		Staff		Students		
	*n*	*n* (%)	*n*	*n* (%)	*n*	*n* (%)	*p*
Gender	1030		663		367		0.08
Female		631 (61.3)		393 (59.3)		238 (64.9)	
Male		399 (38.7)		270 (40.7)		129 (35.1)	
Age (years) *	1030	36.0 (24.0–47.0)	663	44.0 (36.0–52.0)	367	23.0 (21.0–26.0)	<0.05
Highest level of education	1030		663		367		<0.05
High School or lower		209 (20.3)		22 (3.3)		187 (51.0)	
Bachelor’s degree		206 (20.0)		157 (23.7)		49 (13.4)	
University		615 (59.7)		484 (73.0)		131 (35.7)	
Body Mass Index (BMI) (kg/m^2^)	1030	23.4 (21.2–26.4)	663	24.1 (21.9–27.1)	367	22.3 (20.2–24.8)	<0.05
Number of chronic diseases	1030	0 (0–0)	663	0 (0–1.0)	367	0 (0–0)	<0.05
Health literacy	1029	7.0 (0–20.0)	662	7.0 (0–18.0)	367	9.0 (0–20.0)	<0.05
COVID-19 infection (prior to questionnaire)	1030		663		367		0.34
No		788 (76.5)		501 (75.6)		287 (78.2)	
Yes		242 (23.5)		162 (24.4)		80 (21.8)	
COVID-19 infection (prior to booster vaccination)	1012		658		354		0.21
No		669 (66.1)		444 (67.5)		225 (63.6)	
Yes		343 (33.9)		214 (32.5)		129 (36.4)	
Primo-vaccination intention (end of 2020)	1029	90.0 (51.0–100)	662	90.0 (60.0–100)	367	85.0 (49.0–100)	<0.05
Primo-vaccination intention (enrollment in SARSSURV study)	1029	100 (80.0–100)	662	100 (85.0–100)	367	100 (79.0–100)	<0.05
Type of vaccine (dose 1)	1027		662		365		<0.05
AstraZeneca		141 (13.7)		127 (19.2)		14 (3.8)	
Moderna		74 (7.2)		42 (6.3)		32 (8.8)	
PfizerBioNTech		781 (76.0)		465 (70.2)		316 (86.6)	
Johnson & Johnson		31 (3.0)		28 (4.2)		3 (0.8)	
Type of vaccine (dose 2)	1002		639		363		<0.05
AstraZeneca		138 (13.8)		124 (19.4)		14 (3.9)	
Moderna		75 (7.5)		43 (6.7)		32 (8.8)	
PfizerBioNTech		788 (78.6)		471 (73.7)		317 (87.3)	
Johnson & Johnson		1 (0.1)		1 (0.2)		0 (0)	
Vaccination Symptoms	930		634		296		<0.05
Asymptomatic		109 (11.7)		91 (14.4)		18 (6.1)	
Mild		209 (22.5)		153 (24.1)		56 (18.9)	
Moderate		421 (45.3)		276 (43.5)		145 (49.0)	
Severe		191 (20.5)		114 (18.0)		77 (26.0)	
Neutralizing antibody level (prior to questionnaire)	773	40.0 (20.0–160.0)	607	40.0 (20.0–160.0)	166	80.0 (40.0–160.0)	<0.05
Neutralizing antibody level (prior to booster vaccination)	962	80.0 (20.0–160.0)	641	40.0 (20.0–160.0)	321	80.0 (40.0–320.0)	<0.05
P50 (P25–P75)							

Abbreviations: *n*, number; *p*, *p*-value; significant *p*-value < 0.05.

**Table 2:** Perceptions of the COVID-19 booster vaccination among the 1030 study participants.

	All		Staff		Students	
Variable	*n*	*n* (%)	*n*	*n* (%)	*n*	*n* (%)
Acceptance of the COVID-19 Booster Vaccination	1030		663		367	
Self-protection		571 (55.4)		390 (58.8)		181 (49.3)
Protecting others		221 (21.5)		150 (22.6)		71 (19.3)
Compliance with the vaccination strategy		173 (16.8)		92 (13.9)		81 (22.1)
No reason		25 (2.4)		6 (0.9)		19 (5.2)
Efficacy		24 (2.3)		15 (2.3)		9 (2.5)
Geographical and financial accessibilities		15 (1.5)		9 (1.4)		6 (1.6)
Trust in sources of information		1 (0.1)		1 (0.2)		0 (0.0)
Refusal of the COVID-19 Booster Vaccination	1030		663		367	
No reason		354 (34.4)		233 (35.1)		121 (33.0)
Possible post-vaccination side effects		245 (23.8)		169 (25.5)		76 (20.7)
Disagreement with the vaccination strategy		113 (11.0)		60 (9.0)		53 (14.4)
Lack of efficacy		111 (10.8)		78 (11.8)		33 (9.0)
Mistrust of the sources of information		63 (6.1)		34 (5.1)		29 (7.9)
Sufficient protection		62 (6.0)		41 (6.2)		21 (5.7)
Does not contribute to herd immunity		39 (3.8)		19 (2.9)		20 (5.4)
Contraindication		30 (2.9)		25 (3.8)		5 (1.4)
Lack of geographical and financial accessibilities		13 (1.3)		4 (0.6)		9 (2.5)

Abbreviation: *n*, number.

**Table 3:** Results of the univariate and multivariate analyses of the factors influencing the COVID-19 booster vaccination intention among staff members.

			Univariate		Multivariate (*n* = 662)		
	*n*	No vs. Yes	Hesitancy vs. Yes	*p*	No vs. Yes	Hesitancy vs. Yes	*p*
Variable		OR (95% CI)	OR (95% CI)		OR (95% CI)	OR (95% CI)	
Gender	663			0.48			
Female (vs. male)		1.42 (0.68–2.99)	1.30 (0.70–2.40)				
Age (years)	663	0.97 (0.94–1.01)	0.98 (0.96–1.01)	0.17			
Highest level of education	663			0.29			
Bachelor’s degree (vs. High school and lower)		1.61 (0.20–13.17)	1.17 (0.25–5.49)				
University (vs. High School and lower)		0.92 (0.12–7.22)	0.66 (0.15–2.96)				
Body Mass Index (kg/m^2^)	663	0.97 (0.89–1.06)	0.93 (0.86–1.01)	0.16			
Chronic diseases	663	0.70 (0.35–1.39)	0.72 (0.41–1.26)	0.32			
Health literacy	662	1.00 (0.98–1.02)	1.02 (1.00–1.03)	<0.05			
COVID-19 infection (before questionnaire)	663			0.66			
Yes (vs. no)		1.20 (0.54–2.63)	1.31 (0.69–2.52)				
Primo-vaccination intention (end of 2020)	662	0.98 (0.97–0.99)	0.98 (0.97–0.99)	<0.05	1.02 (1.00–1.05)	0.99 (0.98–1.00)	<0.05
Primo-vaccination intention (at SARSSURV enrollment)	662	0.95 (0.94–0.96)	0.96 (0.95–0.97)	<0.05	0.93 (0.91–0.95)	0.97 (0.95–0.98)	<0.05
Vaccination symptoms	634			0.43			
Mild (vs. asymptomatic)		2.21 (0.45–10.90)	2.11 (0.56–7.87)				
Moderate (vs. asymptomatic)		2.93 (0.66–12.99)	3.05 (0.90–10.36)				
Severe (vs. asymptomatic)		2.11 (0.40–11.14)	1.97 (0.49–7.84)				
Neutralizing antibodies (before questionnaire)	607	1.00 (1.00–1.00)	1.00 (1.00–1.00)	0.76			

Abbreviations: *n*, number; OR, odds ratio; *p*, *p*-value; CI, confidence interval; significant *p*-value < 0.05.

**Table 4:** Results of the univariate and multivariate analyses of the factors influencing the COVID-19 booster vaccination intention among students.

			Univariate		Multivariate (*n* = 367)		
	*n*	No vs. Yes	Hesitancy vs. Yes	*p*	No vs. Yes	Hesitancy vs. Yes	*p*
Variable		OR (95% CI)	OR (95% CI)		OR (95% CI)	OR (95% CI)	
Gender	367			0.75			
Female (vs. male)		1.18 (0.58–2.38)	1.26 (0.63–2.54)				
Age (years)	367	1.00 (0.95–1.05)	0.98 (0.92–1.04)	0.80			
Highest level of education	367			0.72			
Bachelor’s degree (vs. High school and lower)		0.80 (0.29–2.25)	0.92 (0.35–2.42)				
University (vs. High School and lower)		0.68 (0.33–1.43)	0.65 (0.31–1.37)				
Body Mass Index (kg/m^2^)	367	0.98 (0.90–1.06)	0.97 (0.89–1.05)	0.65			
Chronic diseases	367	0.77 (0.34–1.74)	0.96 (0.48–1.93)	0.82			
Health literacy	367	1.01 (0.99–1.03)	1.02 (1.01–1.04)	<0.05	1.00 (0.98–1.02)	1.02 (1.01–1.04)	<0.05
COVID-19 infection (before questionnaire)	367			0.46			
Yes (vs. no)		0.59 (0.24–1.46)	0.78 (0.35–1.77)				
Primo-vaccination intention (end of 2020)	367	0.96 (0.95–0.97)	0.98 (0.97–0.99)	<0.05	0.98 (0.96–0.99)	0.98 (0.97–0.99)	<0.05
Primo-vaccination intention (at SARSSURV enrollment)	367	0.96 (0.94–0.97)	0.97 (0.96–0.99)	<0.05	0.97 (0.96–0.99)	0.99 (0.97–1.00)	<0.05
Vaccination symptoms	296			0.74			
Mild (vs. asymptomatic)		0.42 (0.08–2.08)	2.18 (0.25–19.25)				
Moderate (vs. asymptomatic)		0.72 (0.19–2.77)	1.33 (0.16–11.07)				
Severe (vs. asymptomatic)		0.54 (0.12–2.33)	2.07 (0.24–17.66)				
Neutralizing antibodies (before questionnaire)	166	0.98 (0.96–1.00)	1.00 (1.00–1.00)	0.19			

Abbreviations: *n*, number; OR, odds ratio; *p*, *p*-value; CI, confidence interval; significant *p*-value < 0.05.

**Table 5:** Results of the univariate and multivariate analyses of the factors influencing the change between the intention and final decision regarding the COVID-19 booster vaccination among the hesitant staff members.

		Univariate		Multivariate (*n* = 47)	
		Yes vs. No		Yes vs. No	
Variable	*n*	OR (95% CI)	*p*	OR (95% CI)	*p*
Gender	48		0.54		
Female (vs. male)		1.47 (0.43–5.00)			
Age (years)	48	1.04 (0.97–1.11)	0.24		
Highest level of education	48		0.87		
Bachelor’s degree (vs. High school and lower)		1.67 (0.09–31.87)			
University (vs. High School and lower)		2.00 (0.11–35.41)			
Body Mass Index (kg/m^2^)	48	1.08 (0.90–1.31)	0.41		
Chronic diseases	48	1.49 (0.46–4.83)	0.51		
Health literacy	48	0.98 (0.96–1.01)	0.18		
COVID-19 infection (before booster vaccination)	48		<0.05	0.06 (0.01–0.30)	<0.05
Yes (vs. no)		0.06 (0.01–0.29)			
Primo-vaccination intention (end of 2020)	48	1.01 (0.99–1.03)	0.34		
Primo-vaccination intention (at SARSSURV enrollment)	48	1.00 (0.98–1.02)	0.91		
Vaccination symptoms	45		0.59		
Mild (vs. asymptomatic)		4.67 (0.30–73.38)			
Moderate (vs. asymptomatic)		5.14 (0.40–66.15)			
Severe (vs. asymptomatic)		2.67 (0.16–45.14)			
Neutralizing antibodies (before booster vaccination)	47	1.00 (0.99–1.00)	<0.05		

Abbreviations: *n*, number; OR, odds ratio; *p*, *p*-value; CI, confidence interval; significant *p*-value < 0.05.

**Table 6:** Results of the univariate and multivariate analyses of the factors influencing the change between the intention and final decision regarding the COVID-19 booster vaccination among the hesitant students.

		Univariate	
		Yes vs. No	
Variable	*n*	OR (95% CI)	*p*
Gender	40		0.42
Female (vs. male)		2.00 (0.37–10.75)	
Age (years)	40	1.01 (0.86–1.19)	0.87
Highest level of education	40		0.94
Bachelor’s degree (vs. High school and lower)		/	
University (vs. High School and lower)		1.39 (0.23–8.51)	
Body Mass Index (kg/m^2^)	40	0.95 (0.76–1.18)	0.62
Chronic diseases	40	1.24 (0.17–9.31)	0.83
Health literacy	40	1.00 (0.97–1.04)	0.81
COVID-19 infection (before booster vaccination)	40		0.24
Yes (vs. no)		0.36 (0.07–1.99)	
Primo-vaccination intention (end of 2020)	40	1.02 (1.00–1.06)	0.05
Primo-vaccination intention (at SARSSURV enrollment)	40	1.02 (1.00–1.05)	0.09
Vaccination symptoms	28		0.99
Mild (vs. asymptomatic)		/	
Moderate (vs. asymptomatic)		/	
Severe (vs. asymptomatic)		/	
Neutralizing antibodies (before booster vaccination)	35	1.00 (1.00–1.00)	0.48

Abbreviations: *n*, number; OR, odds ratio; /, Quasi-complete separation of data points; *p*, *p*-value; CI, confidence interval; significant *p*-value < 0.05.

**Table 7:** Univariate and multivariate analyses of the factors influencing the change between the intention and final decision regarding the COVID-19 booster vaccination among staff members who changed their mind.

		Univariate			Multivariate (*n* = 593)		
	*n*	No->Yes vs. Maintenance	Yes->No vs. Maintenance	*p*	No->Yes vs. Maintenance	Yes->No vs. Maintenance	*p*
Variable		OR (95%CI)	OR (95%CI)		OR (95%CI)	OR (95%CI)	
Gender	610			0.06			
Female (vs. male)		7.35 (0.94–57.82)	1.84 (0.80–4.25)				
Age (years)	610	1.00 (0.94–1.05)	0.97 (0.93–1.00)	0.16			
Highest level of education	610			0.79			
Bachelor’s degree (vs. high school and lower)		0.41 (0.04–4.15)	0.68 (0.08–6.17)				
University (vs. high school and lower)		0.30 (0.04–2.56)	0.94 (0.12–7.37)				
Body Mass Index (kg/m^2^)	610	1.04 (0.93–1.17)	1.01 (0.93–1.09)	0.76			
Chronic diseases	610	0.80 (0.26–2.40)	1.12 (0.62–2.02)	0.85			
Health literacy	609	0.98 (0.93–1.03)	0.98 (0.95–1.01)	0.36			
COVID-19 infection (prior to booster)	610			<0.05			<0.05
Yes (vs. no)		0.95 (0.25–3.61)	21.04 (6.27–70.65)		0.67 (0.14–3.11)	10.07 (2.75–36.89)	
Primo-vaccination intention (end of 2020)	609	0.98 (0.97–1.00)	0.99 (0.98–1.00)	<0.05			
Primo-vaccination intention (at SARSSURV enrollment)	609	0.97 (0.95–0.98)	0.98 (0.96–0.99)	<0.05	0.96 (0.95–0.98)	0.99 (0.97–1.00)	<0.05
Vaccination symptoms	584			1.00			
Mild (vs. asymptomatic)		/	1.11 (0.31–3.89)				
Moderate (vs. asymptomatic)		/	1.00 (0.31–2.23)				
Severe (vs. asymptomatic)		/	1.07 (0.28–4.12)				
Neutralizing antibody level (before booster vaccination)	594	1.00 (1.00–1.00)	1.00 (1.00–1.00)	<0.05	1.00 (1.00–1.00)	1.00 (1.00–1.00)	<0.05

Abbreviations: *n*, number; OR, odds ratio; /, Quasi-complete separation of data points; *p*, *p*-value; CI, confidence interval; significant *p*-value < 0.05.

**Table 8:** Univariate and multivariate analyses of the factors influencing the change between the intention and final decision regarding the COVID-19 booster vaccination among students who changed their mind.

		Univariate			Multivariate (*n* =286)		
	*n*	No->Yes vs. Maintenance	Yes->No vs. Maintenance	*p*	No->Yes vs. Maintenance	Yes->No vs. Maintenance	*p*
Variable		OR (95% CI)	OR (95% CI)		OR (95% CI)	OR (95% CI)	
Gender	314			0.62			
Female (vs. male)		1.63 (0.50–5.26)	1.22 (0.64–2.34)				
Age (years)	314	1.00 (0.93–1.09)	1.01 (0.96–1.05)	0.97			
Highest level of education	314			0.92			
Bachelor’s degree (vs. high school and lower)		/	0.68 (0.24–1.90)				
University (vs. high school and lower)		1.27 (0.44–3.62)	1.10 (0.57–2.12)				
Body Mass Index (kg/m^2^)	314	0.94 (0.82–1.09)	0.96 (0.89–1.04)	0.49			
Chronic diseases	314	0.84 (0.24–2.94)	0.98 (0.50–1.90)	0.96			
Health literacy	314	0.99 (0.96–1.03)	1.00 (0.99–1.02)	0.84			
COVID-19 infection (prior to booster)	314			<0.05			
Yes (vs. no)		0.69 (0.21–2.24)	2.77 (1.48–5.17)				
Primo-vaccination intention (end of 2020)	314	0.98 (0.96–0.99)	1.00 (0.99–1.01)	<0.05	0.97 (0.96–0.99)	1.00 (0.99–1.01)	<0.05
Primo-vaccination intention (at SARSSURV enrollment)	314	0.98 (0.97–1.00)	1.00 (0.99–1.02)	0.05			
Vaccination symptoms	261			0.92			
Mild (vs. asymptomatic)		0.65 (0.05–7.77)	0.54 (0.11–2.58)				
Moderate (vs. asymptomatic)		0.95 (0.11–8.25)	0.60 (0.15–2.34)				
Severe (vs. asymptomatic)		0.49 (0.04–5.83)	0.82 (0.20–3.40)				
Neutralizing antibody level (before booster vaccination)	286	1.00 (0.99–1.00)	1.00 (1.00–1.00)	<0.05	1.00 (0.99–1.00)	1.00 (1.00–1.00)	<0.05

Abbreviations: *n*, number; OR, odds ratio; /, Quasi-complete separation of data points; *p*, *p*-value; CI, confidence interval; significant *p*-value < 0.05.

## Data Availability

The data are not publicly available due to privacy restrictions.

## Supplementary Materials

The following supporting information can be downloaded at: www.mdpi.com/article/10.3390/vaccines10091485/s1, File S1: statistical analysis with integration of interactions.
