# Supplementary material for: The Dynamic Relationship between the Intention and Final Decision for the COVID-19 Booster: A Study among Students and Staff at the University of Liège, Belgium
Source: Vaccines (Basel). 2022 Sep 6;10(9):1485. doi: 10.3390/vaccines10091485 (PMC9501467; doi:10.3390/vaccines10091485)
Supplement: Supplementary file 1 [file vaccines-10-01485-s001.zip › vaccines-1871099-supplementary.pdf]

Appendix 1: statistical analysis with integration of interactions

1. *Results of univariate and multivariate analyses of factors influencing COVID-19 booster vaccination intention among staff members*

Multivariate forward stepwise logistic regression found that past COVID-19 vaccination intention was predictor of booster vaccination intention among staff members. Interactions analyses found that COVID-19 vaccination intention at the end of 2020 with at the time of SARSSURV study enrollment were significantly associated with booster intention ($p<0.05$) (Table S1). However, the main message was unchanged.

Table S1. Interactions between significant variables of final model for COVID-19 booster vaccination intention among staff members

	Interactions (N=662)			
	No vs Yes		Hesitancy vs Yes	
**Variable**	**Estimate ± SE**	**P**	**Estimate ± SE**	**P**
Primo-vaccination intention (end of 2020)	0.07 ± 0.02	<0.05	0.04 ± 0.02	<0.05
Primo-vaccination intention (at SARSSURV enrollment)	-0.05 ± 0.01	<0.05	-0.02 ± 0.01	0.08
Primo-vaccination intention (end of 2020)* Primo-vaccination intention (at SARSSURV enrollment)	-0.00 ± 0.00	<0.05	-0.00 ± 0.00	<0.05

Abbreviations : N, number; SE, standard error; P, p-value; significant p-value <0.05

2. *Results of univariate and multivariate analyses of factors influencing COVID-19 booster vaccination intention among students*

Multivariate forward stepwise logistic regression found that health literacy and past COVID-19 vaccination intention was predictor of booster vaccination intention among students. However, the quality of the model is better for the model without the interactions (AIC=422.99) compared to the model with the interactions (AIC=428.77).

3. *Results of univariate and multivariate analyses of the factors influencing change between intention and final decision regarding COVID-19 booster vaccination among the hesitant staff members*

After multivariate analysis, only one variable was significantly associated with change between intention and the final decision regarding the COVID-19 booster. Thus, no interaction could be tested.

4. *Results of univariate and multivariate analyses of the factors influencing change between intention and final decision regarding COVID-19 booster vaccination among the hesitant students*

Univariate analyses found no significant variable. Thus, no interaction could be tested.

5. *Univariate and multivariate analyses of factors influencing change between intention and final decision regarding COVID-19 booster vaccination among staff who changed their mind*

Multivariate analysis showed that COVID-19 infection, past COVID-19 vaccination intention at SARSSURV enrollment, and neutralizing antibody level were significantly associated with a change between intention and final decision regarding COVID-19 booster vaccination. Interactions analyses found no significant variable (Table S2). By comparing model, the best model keeps only the interaction between intention and covid (AIC=280.974). The model included all interactions presented a higher AIC (AIC=284.811). However, the main message was unchanged.

Table S2. Interactions between significant variables of final model for staff members who changed their mind

	Interactions (N=593)			
	No->Yes vs maintenance		Yes->No vs maintenance	
**Variable**	**Estimate ± SE**	**P**	**Estimate ± SE**	**P**
COVID-19 infection (prior to booster vaccination- Yes vs No)	-3.66 ± 2.20	0.10	-0.21 ± 1.54	0.89
Primo-vaccination intention (at SARSSURV enrollment)	-0.05 ± 0.01	<0.05	-0.04 ± 0.02	<0.05
Neutralizing antibody level (before booster vaccination)	-0.00 ± 0.00	0.79	0.00 ± 0.00	<0.05
Primo-vaccination intention (at SARSSURV enrollment)* COVID-19 infection	0.05 ± 0.03	0.07	0.03 ± 0.02	0.10

Abbreviations : N, number; SE, standard error; P, p-value; significant p-value <0.05

6. *Univariate and multivariate analyses of factors influencing change between intention and final decision regarding COVID-19 booster vaccination among students who changed their mind*

Multivariate analysis showed that past COVID-19 vaccination intention at the end of 2020, and neutralizing antibody level were significantly associated with a change between intention and final decision regarding COVID-19 booster vaccination. Interactions analyses found that COVID-19 vaccination intention at the end of 2020 and neutralizing antibody level were significantly associated with a change between intention and final decision regarding COVID-19 booster vaccination (Yes->No) (p<0.05) (Table S3). However, we decided to keep the results without the interactions because in general, odds ratios of interaction term are complicated to interpret. This makes the message harder to get across (health promotion article).

Table S3. Interactions between significant variables of final model for students who changed their mind

	Interactions (N=286)			
	No->Yes vs maintenance		Yes->No vs maintenance	
**Variable**	**Estimate ± SE**	**P**	**Estimate ± SE**	**P**
Primo-vaccination intention (end of 2020)	-0.03 ± 0.01	<0.05	-0.01 ± 0.01	0.12
Neutralizing antibody level (before booster vaccination)	-0.00 ± 0.00	0.14	-0.00 ± 0.00	0.41
Primo-vaccination intention (end of 2020)* Neutralizing antibody level (before booster vaccination)	0.00 ± 0.00	0.60	0.00 ± 0.00	0.01

Abbreviations : N, number; SE, standard error; P, p-value; significant p-value <0.05
